# Effects of Increasing the Negativity of Implicit Outcome Expectancies on Internet Gaming Impulsivity

**DOI:** 10.3389/fpsyt.2020.00336

**Published:** 2020-04-29

**Authors:** Shumeng Hou, Xiaoyi Fang, Nan Zhou, Pengpeng Cai

**Affiliations:** ^1^Harbin Institute of Technology, Shenzhen, Shenzhen, China; ^2^Institute of Developmental Psychology, Beijing Normal University, Beijing, China; ^3^Faculty of Education, Beijing Normal University, Beijing, China

**Keywords:** problem Internet gaming, evaluative conditioning, delay discounting, college students, Go/No-Go Association task

## Abstract

Outcome expectancies have been found to play important roles in addictive behaviors. Research has shown that implicit outcome expectancies (OE) were significantly correlated with Internet gaming behaviors among players with Internet gaming disorder (IGD). However, few empirical studies have further examined the relationship between implicit OE and players with IGD. This study first strengthened the implicit association between Internet games and negative outcomes using an evaluative conditioning paradigm (EC) and then examined the effects of increasing the negativity of implicit OE on Internet gaming impulsivity. Thirty-nine college students who were diagnosed as players with IGD participated in the study. Manipulation checks showed that after the EC was introduced, participants associated Internet gaming stimuli more closely with negative outcomes than with positive outcomes. After the implicit OE were effectively altered to be negative, players with IGD performed better in the delay discounting paradigm, showing a lower impulsivity with respect to Internet gaming.

## Introduction

Internet gaming has become a dominant leisure activity of young people's life (1). In a recent report from the China Internet Network Information Center, more than half (58.4%) of the overall Internet users in China are active gamers (2). However, the excessive use of Internet games can lead to an Internet gaming disorder (IGD) (3) that can cause severe damage to young people's mental health and social functioning (4–7). Correspondingly, a diagnosis of IGD has been formally introduced into the 11th revision of International Classification of Diseases (ICD-11) and the fifth edition of Diagnostic and Statistical Manual of Mental Disorders (DSM-V) as a disorder (8, 9).

Like people who suffer from substance dependence or pathological gambling, the most important trait of gamers with IGD is believed to be impulsivity. Researchers believe that IGD is related to the disposition to engage in rash and spontaneous behavior without concern for the risks or the negative future consequences associated with such behavior (10–12). Gamers with IGD not only demonstrated higher self-reported impulsivity in questionnaires (13, 14) but were also less able to control their impulses in behavioral experiments. Using the delay discounting paradigm, adolescents with IGD were found to be more likely to overvalue immediate outcomes and neglect future outcomes, suggesting that impulsivity may be a behavioral marker of IGD (15).

Some researchers believe that the high impulsivity of young adults who engage in addictive behaviors, despite warnings concerning the dangers of such behaviors, might be related to the general tendency for people to have anticipated positive consequences for such actions that outweigh the possible negative outcomes of those actions (16). Thus, an individual's decision to initiate and maintain addictive behaviors likely reflects their differential salience with respect to what they expect to gain from those behaviors (i.e., positive outcome expectancies, positive OE) versus what they expect to lose as a result of those behaviors (i.e., negative outcome expectancies, negative OE) (17). For example, young adults' gambling behaviors could be predicted by their anticipation of positive gambling outcomes even though gambling behaviors have been found to be negatively correlated with negative OE (18). Researchers also found consistent positive relationships between positive OE and Internet usage (19, 20). However, the results regarding negative OE are far from consistent (13, 21, 22). Some researchers have found a negative correlation between negative OE and Internet usage (19), while in other studies, negative OE failed to demonstrate a significant negative effect on Internet use (21, 22).

These contradictory findings might be because most previous research used self-reported questionnaires, which assessed participants' explicit, conscious thoughts about the possible outcomes of Internet usage. However, unlike goal-directed actions, addictive behaviors may not depend on awareness but instead may be directly triggered by conditioned stimuli with no recruitment of higher cognitive processes being involved in the initiation of such behaviors (23). It has been shown that the initiation of drug seeking may depend solely upon implicit mechanisms, without an individual's explicit intention or choice processes being involved in initiating such behavior (23–25). Although research is still rare regarding the possible relationship between OE and behaviors of IGD, a recent study has found that gamers with IGD hold different explicit and implicit OE (26). Specifically, young gamers with IGD reported negative explicit OE, while they still associated Internet gaming more closely with positive outcomes in the Go/No-go association task (27), suggesting that such gamers with IGD have relatively positive implicit Internet gaming outcome expectancies (OEs) (26). In addition, only implicit Internet gaming OEs were found to be significantly correlated with the number of years that gamers with IGD persist in playing games (26). In this sense, the unconscious anticipation of the possible outcomes of playing Internet games may play an important role in predicting Internet gaming impulsivity. Thus, in order to clarify the effect of OE on IGD, the current study focuses on evaluating the impact of implicit OE on Internet gaming impulsivity among players of IGD.

Although, to our knowledge, no empirical study has examined the specific effects of altering implicit Internet gaming OEs on IGD, several research papers regarding substance abuse have already attempted to produce interventions with respect to alcohol consumption by directly manipulating implicit alcohol consumption OEs (28, 29). Some studies have demonstrated that changing implicit cognition can effectively reduce people's alcohol consumption (30). For example, when college students were semantically primed with positive outcomes such as “funny”, “relax”, or “talkative”, such positive priming resulted in significantly higher rates of beer consumption; whereas when students were semantically primed with negative outcomes such as “sick”, “dizzy”, or “problem”, such negative priming resulted in significantly lower rates of beer consumption (31). In contrast, when participants were asked to consciously change their OEs toward drinking (e.g., when participants were asked to reword their positive OE of “Alcohol makes me worry less” to “I seem to be worried less when I'm out with my friends, regardless of whether or not I have been drinking”), their rate of alcohol consumption failed to change (32). Previous research also used Evaluative Conditioning (EC) (33, 34) in changing implicit attitudes or beliefs. During the EC paradigm, participants were forced to associate target terms with positive or negative stimuli implicitly, and thereby get the positive or negative attribute. It has been documented that EC can have a significant effect in creating and changing implicit attitudes and beliefs (35, 36). Once an implicit attitude has been created or changed by EC, such changes can last at least 24 hours (37, 38). In fact, EC has already been used successfully to intervene in problem behaviors such as unhealthy eating habits (39). However, since the EC paradigm has not been adopted in studies looking to provide interventions for IGD it is not known whether implicit Internet gaming OE can also be changed through the use of EC. Nor do we know whether changes in implicit OE will have an influential effect on the Internet gaming impulsivity of problematic Internet gamers.

In summary, the current study attempted to address several limitations of past studies. First, in order to clarify the effect of Internet gaming OE on Internet gaming impulsivity, we distinguished implicit OE from explicit OE. The present study merely focuses on the unconscious beliefs about the possible outcomes of playing Internet games among players with IGD. The Go/No-go association task (27) was adopted in evaluating the implicit OE of players with IGD (26). Second, limited existing research regarding implicit Internet gaming OE only used correlation analysis in exploring the relationship between implicit Internet gaming OE and IGD. In the present study, we went further by examining the possible causal effects of changing implicit Internet gaming OE on Internet gaming impulsivity among players with IGD. Specifically, the current study first adopted the EC paradigm to enhance the association of Internet gaming with negative outcomes and to thereby increase the negativity of implicit Internet gaming OEs among students with IGD. After the manipulating effect was indicated, the study further discussed the effects of increasing the negativity of implicit Internet gaming OE on the Internet gaming impulsivity of gamers with IGD.

## Methods

### Participants

Ninety male college students from three universities in Beijing completed the Chinese Internet Addiction Scale (CIAS) (40). Twenty-five of them participated in the pilot study. Of the remaining 65 students, 39 met the following two criteria and were categorized as players with IGD: first, their scores on CIAS were higher than 63, an indicator considered most likely to diagnose one as an Internet addict (41); second, their online behaviors fulfilled all of the following three criteria: (1) their main reason for accessing the Internet was to play Internet games; (2) the games they played belong to the game types categorized by the National Internet Developmental Situation Report of China (42); and (3) they had spent approximately 20 or more hours playing Internet games each week over the past two months. Among the 39 participants, 29 (74.4%) were undergraduates and 10 (25.6%) were postgraduate students. The study was approved by the Ethics Committee of the University where the study was conducted. Students volunteering for the study also signed consent forms before participating in the study.

### Measures

#### Implicit Internet Gaming OEs

We used the Go/No-go Association Task (GNAT) (27) to assess the implicit Internet gaming OEs of study participants. Implicit Internet gaming OEs were represented by the association that people had between Internet gaming words and positive or negative outcomes during the GNAT (26, 27). In the GNAT (27), the strength of such associations was assessed by the degree to which items belonging to the target category and attribute (e.g., fruit and good) could be distinguished from distracter items that did not belong to those concepts (e.g., bad). Two conditions were included in the GNAT. One condition required the simultaneous identification of stimuli that represented the target category (e.g., fruit) and an attribute (e.g., good); a second condition required the simultaneous identification of stimuli that represented the same target category (e.g., fruit) and an alternative attribute (e.g., bad). In both conditions, participants' response latencies were recorded as the dependent variable. The extent to which fruit is associated with good versus bad was reflected in the response latencies involved in associating fruit with one evaluative attribute (e.g., good) as opposed to the other (e.g., bad).

In the present study, players with IGD completed a word-based GNAT task, which presented 50 stimuli words with three to four Chinese characters. These same stimuli were used and proven to be effective in Hou and Fang's study (26). The category words used are Internet gaming words, and the attribute words used are positive or negative outcome words. Specifically, there were 10 Internet gaming words that are neutral nouns (e.g., game currency “游戏币 you2 xi4 bi4”), 10 direct positive and negative outcome words that could be obtained directly from the game itself (e.g., level up “升级了 sheng1 ji2 le”; lose game “输比赛shu1 bi3 sai4”), and 10 indirect positive and negative outcome words that might reflect the real life situations of individuals after they had played Internet games (e.g., happy “高兴的 gao1 xing4 de”; poor academic results “学业差 xue2 ye4 cha4”) (See details in **Appendix 1** in the **Supplementary Material**). The GNAT included four blocks, with 50 items in each block. Participants were asked to associate Internet gaming words with the appropriate outcome words in each block. The effects were calculated using the average response time (in milliseconds) of instances where participants correctly associated Internet gaming words with the appropriate outcome words within each block. Formal study started only after five practice trials to make sure that participants had fully understood the instructions for completing the task.

#### Internet Gaming Impulsivity

**Figure 1:**
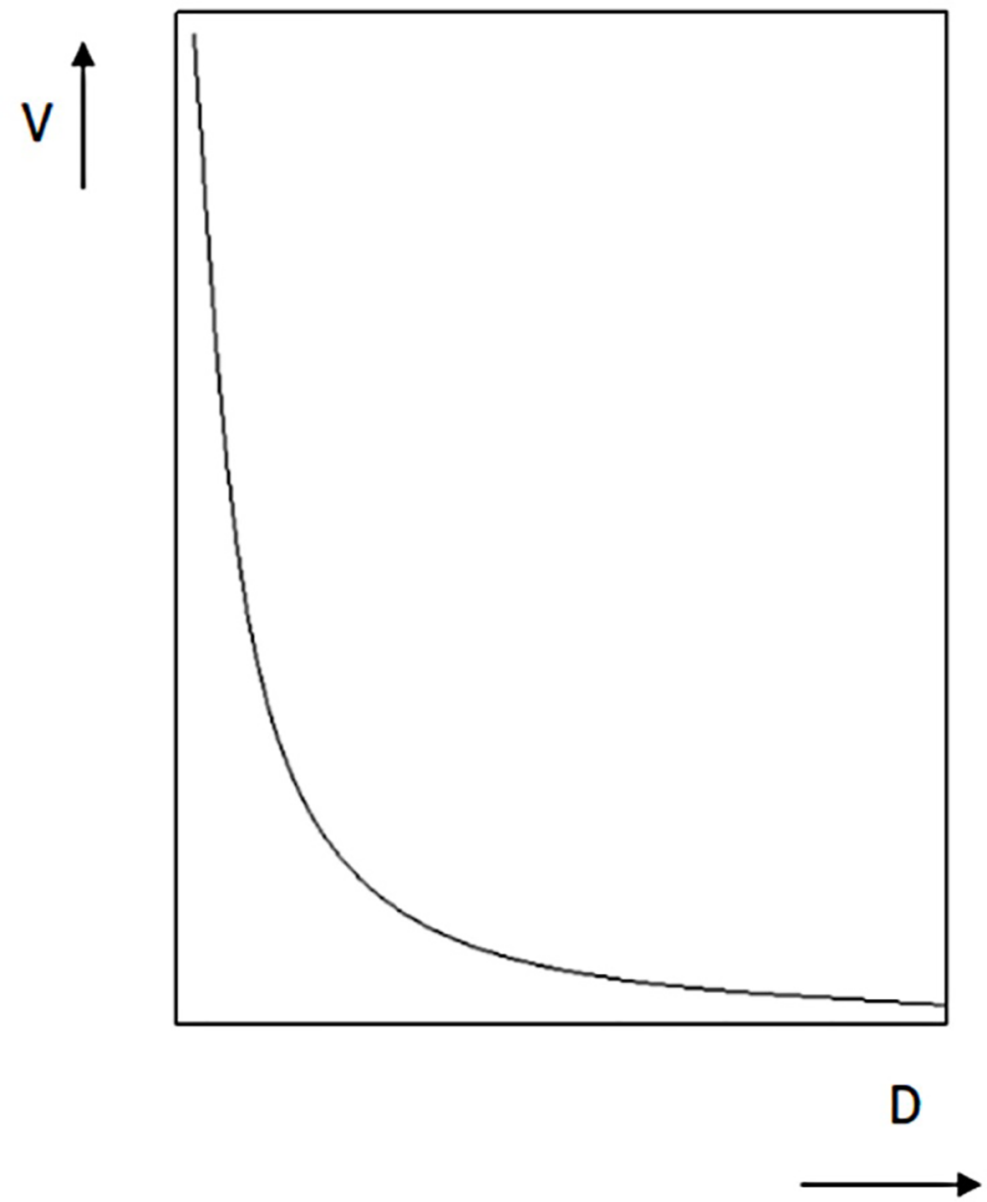
Hyperbolic delay discount functions.

Internet gaming impulsivity was assessed using the delay discounting paradigm (15). The delay discounting paradigm is an amount-adjusting computerized choice procedure. Participants were asked to make a series of choices between an immediate smaller reward and a delayed larger reward. The data in delay discounting typically fits Mazur's (42) hyperbolic equation: V = A/(1 + kD). A larger k value represents greater discounting, meaning that a delayed reward loses subjective value more quickly over time. Thus, the k value is used as an index of impulsivity (**Figure 1**)

In the classical delay discounting paradigm, the reward used was money (43). Later, researchers studying addictive behaviors changed the rewards to stimuli that participants were addicted to (44). Accordingly, in the present study, *hours of playing Internet games*, instead of money, were used as rewards in the delay discounting paradigm. All the participants with IGD were asked to estimate the duration of time spent playing Internet games that would be *equivalent to receiving a given amount of money* before the formal experiment began (44). The magnitude of the reward given was the number of hours of playing Internet games that was deemed to be equivalent to $100 by each participant. The delays used in the study were 2 days, 1 month, and half a year.

Specifically, participants would read the following instructions in the first session:

“I want you to imagine that you have a choice between receiving some money and receiving a duration of time to play Internet games. For the following statement, please fill in the number of hours that would make the two choices equally attractive to you:

Receiving RMB 100 right now would be just as attractive as receiving ::_ hours to play Internet games.”

During the second session, participants were asked to make a series of choices between a standard larger, but later, (LL) reward option and an adjusted smaller, but sooner, (SS) reward option. The magnitude of the SS option was adjusted across trials until an indifference point was determined. The formal study started only after five practice trials had been conducted to make sure that participants had fully understood the instructions involved in completing the task. The equation of V = A/(1 + kD) was used to estimate the discounting rate “k” as the index of Internet gaming impulsivity.

#### The Manipulation of Implicit Internet Gaming OEs

**Figure 2:**
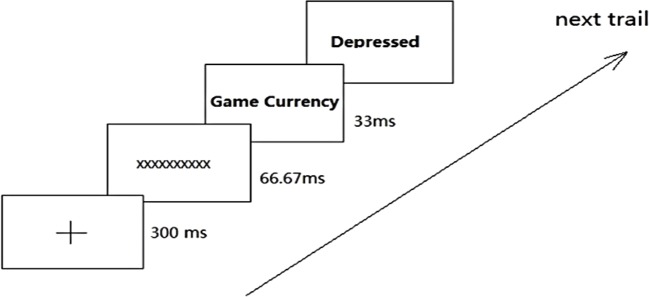
Internet gaming words—negative outcome words—pair in EC.

**Figure 3:**
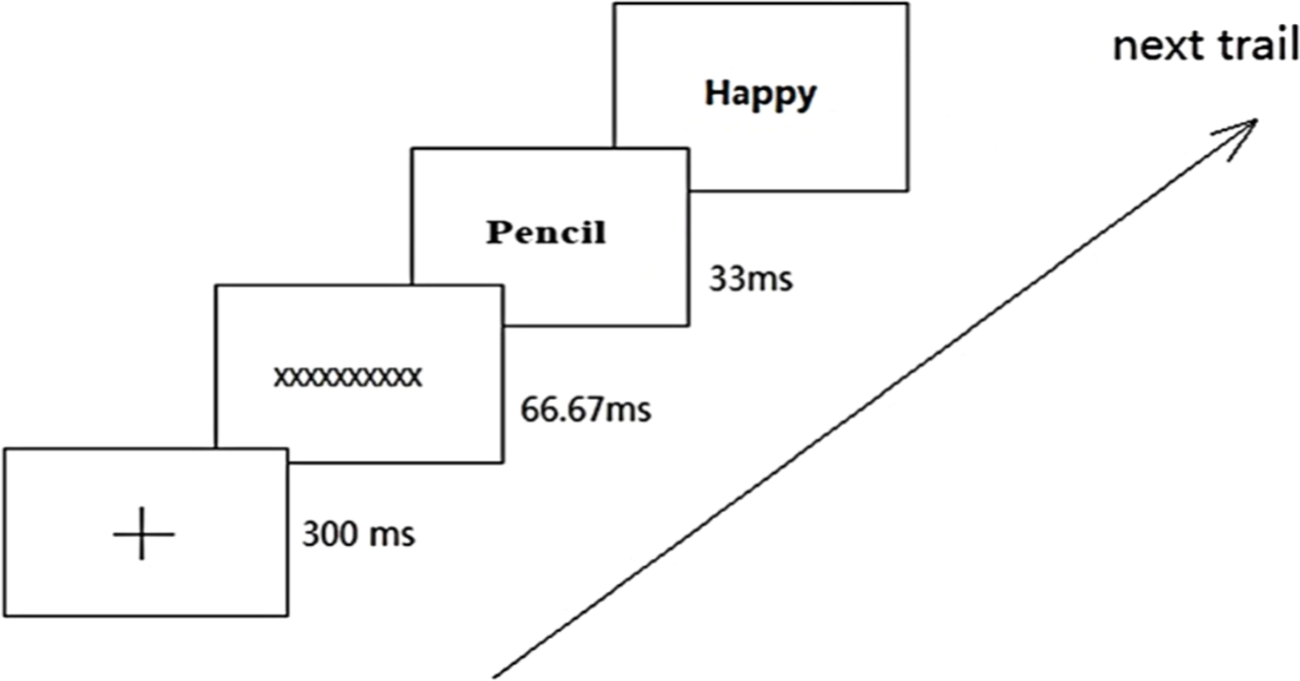
Common life—positive outcome word—pair in EC.

An EC paradigm was used to strengthen the association between Internet gaming and negative outcomes and, thereby, to increase the negativity of implicit Internet gaming OE (35, 36). During the EC paradigm, participants were forced to associate Internet gaming words with negative outcome words implicitly. Specifically, both direct and indirect negative outcomes of playing Internet games were paired with Internet gaming words, and these word pairs were presented in each trial (see details in **Figure 2**). Each Internet gaming word was randomly presented 20 times. Given that there are 10 Internet gaming words and 20 negative-outcome words (10 direct and 10 indirect negative outcome words), there were 200 pairings involving Internet gaming words and negative-outcome words. To make participants concentrate on the experiment, both common-life words and positive-outcome words also appeared in pairs randomly (**Figure 3**). This entire intervention described above takes approximately 20 minutes to complete.

### Procedures

The whole procedure included both pre-tests and post-tests of implicit Internet gaming OE and of impulsivity, and the process used to manipulate participants' implicit Internet gaming OE was based on EC. GNAT was used to investigate implicit Internet gaming OE. A delay discounting paradigm was used to evaluate Internet gaming impulsivity. After the pre-test, players with IGD took a 10-minute rest and then proceeded to the EC program. A post-test was implemented 24 hours later using the same methods as those used in the pre-test. The entire computer-based procedure was supported by an application written in the C++ programming language.

### Statistical Analysis

We checked the changes in the implicit Internet gaming OE of players with players with IGD and the changes in Internet gaming impulsivity under the delay discounting paradigm using repeated measures analysis of variance (ANOVA). All the k values in the delay discounting paradigm were calculated by using a natural log transformation, which normalized the distributions (45). Finally, paired t-test analysis was used to test the changes in Internet gaming impulsivity between the effective and non-effective manipulating groups of implicit Internet gaming OE. All analyses were performed with IBM SPSS Statistics v20 for Windows.

## Results

### Manipulation Check of the Changes in Implicit Internet Gaming OEs

We first checked the manipulating effect of the changes in implicit Internet gaming OE among students with IGD. The mean scores of the latent response time for associating Internet gaming words with positive and negative outcome words in the pre-test (T1) and the post-test (T2) are listed in **Table 1**.

**Table 1:** The mean scores of latent response times obtained when associating Internet gaming words with positive and negative outcome words (SD).

	DP		DN		*t* (38)	Cohen's d	IP		IN		*t*(38)	Cohen's d
	*M* (ms)	*SD*	*M* (ms)	*SD*			*M* (ms)	*SD*	*M* (ms)	*SD*		
**T1**	627.80	66.81	647.56	62.43	−2.79*	–0.31	647.56	62.43	651.07	80.62	–0.24	–0.05
**T2**	617.56	76.29	586.96	66.21	2.11*	0.43	590.42	62.20	561.56	50.75	2.58**	0.51

N = 39; DP = latent response time (ms) spent on associating direct positive outcomes with Internet games, with shorter latent response times representing stronger implicit direct positive OE; DN = the latent response time spent on associating direct negative outcomes with Internet games, with shorter latent response times representing stronger implicit direct negative outcome expectancy; IP = latent response time spent on associating indirect positive outcomes with Internet games, with shorter latent response times representing stronger implicit indirect positive outcome expectancy; IN = latent response time spent on associating indirect negative outcomes with Internet games, with shorter latent response times representing stronger implicit indirect negative outcome expectancy.

*p < 0.05, **p < 0.01.

We estimated whether the implicit Internet gaming OE became more negative after performing the EC paradigm. Specifically, we compared the strength of association that players with IGD exhibited when associating Internet gaming words with positive and negative outcomes before and after performing the EC paradigm. From the descriptive analysis mentioned above (see **Table 1**), before EC (T1), the latent response time was shorter when players with IGD associated Internet games with direct positive outcomes than with direct negative outcomes (DP < DN). After EC (T2), the latent response time was shorter when players with IGD associated Internet games with direct negative outcomes than with direct positive outcomes (DP > DN). In addition, the latent response time obtained when players with IGD associated Internet gaming words with indirect positive and negative outcomes was similar in T1. However, in T2, the latent response time obtained when players with IGD associated Internet gaming words with indirect negative outcomes was faster than that with indirect positive outcomes (IP > IN).

We then conducted repeated measures ANOVA with testing time (T1 vs. T2), the attribute of OE (positive vs. negative), and direct or indirect outcomes as within-subject independent variables and defining the latent response time of associating Internet gaming words with outcome words to be the dependent variable. The results showed that the main effect of testing time was significant, *F* (1, 38) = 11.75, *p* < 0.001, *η*^2^ = 0.24; the interaction between testing time and the attribute of implicit Internet gaming OE was also significant, *F* (1,38) = 11.31, *p* < 0.01, *η*^2^ = 0.23. A planned paired t-test showed that in T1, DP associations was stronger than DN, *t* (38) = –2.79, *p* < 0.01, while in T2, DN associations was stronger than DP, *t* (38) = 2.11, *p* < 0.05 (see **Table 1**). Such findings suggest that in T1, players with IGD associated Internet gaming more closely with direct positive outcomes, whereas after participating in the EC paradigm, players with IGD associated Internet gaming more closely with direct negative outcomes. The attribute of implicit direct Internet gaming OE changed from positive to negative. On the other hand, there was no significant difference between IP and IN in T1, *t* (38) = –0.24, *p* = 0.81, but after conducting the EC paradigm, IN associations were significantly stronger than IP associations in T2, *t* (38) = 2.58, *p* < 0.01 (see **Table 1**). This result suggests that in T1, the strength with which players with IGD associated Internet gaming with indirect positive outcomes and with negative outcomes had no significant difference. However, after participating in the EC paradigm, players with IGD associated Internet gaming more closely with indirect negative outcomes than with indirect positive outcomes. Thus, after the EC manipulation, the attribute of indirect implicit Internet gaming OE changed from neutral to negative. Taking into account both direct and indirect implicit Internet gaming OE together, the EC paradigm effectively increased the negativity of implicit Internet gaming OE among students with IGD.

### The Influential Effects of Increasing the Negativity of Implicit Internet Gaming OE on Internet Gaming Impulsivity

We then estimated the influential effect of changing implicit Internet gaming OE on Internet gaming impulsivity among players with IGD. Specifically, we asked whether participants demonstrated lower impulsivity under the delay discounting paradigm after participating in EC. We first compared players' Internet gaming impulsivity before and after participating in the EC paradigm. The results showed that at all delay levels, the k value decreased in T2 compared with that in T1, and the k value also decreased with the increase of the delay time (see **Table 2**). Repeated ANOVA showed that the main effect of testing time was significant, *F* (1, 38) = 7.02, *p* < 0.05, *η*^2^ = 0.16; the main effect of delay level was significant, *F*(2, 76) = 25.18, *p* < 0.001, *η*^2^ = 0.40; and the interaction between testing time and delay level was not significant, *F*(2, 76) = 0.43, *p* = 0.67, *η*^2^ = 0.01. Such findings suggest that after the negativity of implicit Internet gaming OE was increased by participating in the EC paradigm, Internet gaming impulsivity was also reduced significantly in T2 as compared to T1. In addition, Internet gaming impulsivity decreased significantly with the increase of delay levels (see **Table 2**).

**Table 2:** The delay discounting rate (k value) before and after the EC paradigm.

Delay level	T1（N = 39）		T2（N = 39）	
	*k*	*SD*	*k*	*SD*
Two days	1.56	0.89	1.40	1.13
One month	1.01	1.21	0.69	1.07
Half a year	0.63	1.25	0.38	1.23

Furthermore, we asked whether such changes in Internet gaming impulsivity the result of the increased negativity of implicit Internet gaming OE were. We first categorized the participants into two groups depending on whether their attribute of implicit Internet gaming OE was effectively changed to negative by the EC paradigm. Specifically, participants whose attribute of implicit Internet gaming OE turned from positive to negative after participating in the EC paradigm were placed in the effective manipulating group. Participants whose attribute of implicit Internet gaming OE did not change or changed from negative (T1) to positive (T2) after participating in the EC paradigm, were placed in the non-effective manipulating group. When considering direct and indirect implicit Internet gaming OE separately, there were 16 participants in the effective manipulating group of direct OE and 23 participants in the non-effective manipulating group of direct OE. With respect to indirect implicit Internet gaming OE, there were 15 participants in the effective manipulating group of indirect OE and 24 participants in the non-effective manipulating group of indirect OE. The mean scores for the k values of different delay levels in both groups are shown in **Table 3**.

**Table 3:** The mean scores for k values of participants in the effective manipulating groups and the non-effective manipulating groups for both direct and indirect implicit Internet gaming OE.

			T1		T2		*t*	Cohen's d
			*M*	*SD*	*M*	*SD*		
Effective manipulating group	Direct OE (N = 16)	2 days	1.39	0.68	1.14	0.79	1.95*	0.34
		1 month	0.99	1.24	0.64	0.98	2.10*	0.31
		Half a year	0.74	1.47	0.43	1.4	1.12	0.22
	Indirect OE (N = 15)	2 days	1.47	0.58	1.17	0.72	2.72**	0.46
		1 month	0.92	1.03	0.55	0.53	2.00*	0.45
		Half a year	0.64	1.34	0.31	1.25	1.125	0.25
Non-effective manipulating group	Direct OE (N = 23)	2 days	1.67	1.01	1.57	1.3	0.70	0.09
		1 month	1.03	1.21	0.73	1.14	1.75	0.26
		Half a year	0.54	1.10	0.35	1.13	0.83	0.17
	Indirect OE (N = 24)	2 days	1.61	1.04	1.54	1.31	0.62	0.06
		1 month	1.07	1.34	0.78	1.3	1.77	0.22
		Half a year	0.61	1.22	0.43	1.24	0.80	0.15

*p < 0.05, **p < 0.01.

Paired t-tests (see **Table 3**) showed that when the delay level was 2 days, in the effective manipulating group of both direct and indirect implicit OE, the k value significantly decreased in T2 compared with that in T1. However, in the non-effective manipulating group of both direct and indirect implicit OE, the k value showed no significant difference between T2 and T1. Similarly, when the delay level was one month, in the effective manipulating group of both direct and indirect implicit OE, the k value significantly decreased in T2 compared with that in T1. However, in the non-effective manipulating group of both direct and indirect implicit OE, the k value showed no significant difference between T2 and T1. However, when the delay level was half a year, none of effective manipulating group or non-effective manipulating group groups showed significant changes in k value between T1 and T2.

In summary, after participating in the EC paradigm, players with IGD associate Internet gaming more closely with negative outcomes than with positive ones, which suggests a significant increase in the negativity of implicit Internet gaming OE. Accordingly, Internet gaming impulsivity also decreased after players with IGD participated in the EC paradigm. Finally, this decrease in Internet gaming impulsivity was probably due to the increasingly negative implicit Internet gaming OE.

## Discussion

The present study used the EC paradigm to increase the negativity of implicit Internet gaming OE among students with IGD examined the influential effects of such an increase on Internet gaming impulsivity among those same students. The results first supported that after participating in the EC paradigm, students with IGD associate Internet gaming words more closely with negative outcomes than with positive ones, suggesting an increase in the negativity of implicit Internet gaming OE. Moreover, Internet gaming impulsivity among participants decreased after the negativity of participants' implicit OE became stronger. Our study is one of the initial efforts aimed at demonstrating the effect of changing implicit Internet gaming OE on Internet gaming impulsivity among players with IGD based on experimental tasks. The findings of the current study also demonstrated the possibility for inducing a short-term change in Internet gaming impulsivity within a laboratory setting.

### The Effectiveness of Changing Implicit Internet Gaming OEs Using the EC Paradigm

Although research has shown that EC was effective in changing people's attitudes and beliefs (34, 46), little is known about whether EC can be successfully applied in changing attitudes of players with IGD about the outcomes of playing Internet games. Our study was among the initial efforts to demonstrate that EC can change implicit OEs among young people with IGD. Specifically, after associating Internet gaming stimuli with negative outcomes using the EC paradigm, implicit Internet gaming OE among players with IGD became negative. This finding was consistent with previous studies regarding evaluative conditioning, which found that when target stimuli are associated with negative/positive stimuli, the target stimuli's valence will often change in accordance with the negative/positive stimuli it has been paired with (36, 47–49). Accordingly, EC has proven effective in changing people's implicit attitude toward target objects such as fruit, black people, or food (27, 39). Our study expanded on previous research by successfully applying the EC paradigm to changing the valence of implicit attitudes toward a kind of addictive behavior (Internet gaming).

### The Influential Effect of Increasing the Negativity of Implicit OEs on Internet Gaming Impulsivity

While exploring the mechanisms of addiction, research regarding substance abuse has suggested that drug seeking and drug taking habits were reinforced by Pavlovian conditioning. Drug-associated stimuli in the environment act as conditioned reinforcers that facilitate the development of incentive habits (50, 51). Such incentive habit processes may facilitate the transition to compulsivity, the hallmark of addiction (52). Based on this finding, we asked, “If Internet gaming stimuli were associated with negative outcomes, would the impulsivity associated with IGD thereby be inhibited?” Such a hypothesis was supported by the results of the current study. Like substance abuse, the impulsivity of players with IGD may also be reinforced by Pavlovian conditioning. Hence, after participating in EC, where IGD players associated Internet gaming stimuli more closely with negative outcomes, their Internet gaming impulsivity significantly decreased. In addition, participants whose implicit OE toward Internet gaming were significantly changed to be negative showed a significant decrease in their Internet gaming impulsivity, whereas people whose implicit OE of Internet gaming were not effectively changed to be negative showed no change in Internet gaming impulsivity. Such findings provide behavioral evidence about the causal relationship between implicit OEs and Internet gaming impulsivity. Our study on implicit Internet gaming OE also contributed to the understanding of why young people with IGD are unable to resist playing Internet games even when they clearly *know* (explicitly) the negative outcomes of such excessive Internet gaming.

The present study also has practical implications. Previous researchers have applied implicit methods when intervening in problem behaviors such as smoking or alcohol consumption (53–57). The EC paradigm has proven to be effective in providing interventions for phobias, sexual, and food-related problem behaviors (39, 58). However, to our knowledge, our study was among the first to show how one might change the implicit OEs of students' with IGD using the EC paradigm. In addition, we also proved that Internet gaming impulsivity can be inhibited within laboratory settings for at least 24 hours by increasing the negativity of people's implicit Internet gaming OE. It would be worthwhile for future studies to incorporate implicit methods into intervention programs that seek to control the Internet gaming impulsivity of players with IGD.

## Limitations and Future Directions

The present study has several limitations. First, the players with IGD in our study were all college students. Admittedly, our findings may not be generalizable to other groups of people. In future studies, the mechanism underlying these findings may need further discussion with respect to players with IGD of different ages or with different educational backgrounds. Second, the post-test in our study was taken 24 hours after the conducting the EC paradigm. Thus, our study only showed that participating in the EC paradigm may change participants' implicit OEs within a short period of time. The long-lasting effects of EC on the changing implicit OE of participants needs to be verified in future studies by conducting a longitudinal study. Third, the current research used a task-based paradigm to change implicit OEs and thus inhibit Internet gaming impulsivity among players with IGD only in laboratory settings; however, whether the paradigm has long lasting interventional effects on the impulsivity of players with IGD is not known. Future studies may establish a more complete intervention program to be used outside laboratory settings, and such studies may also further examine the possibility of intervening in players with IGD by changing implicit OE.

## Conclusion

This is the first study used behavioral experiments to explore the relationship between implicit Internet gaming OEs and Internet gaming impulsivity. The present study found that when the negativity of implicit Internet gaming OE was increased, the Internet gaming impulsivity of gamers with IGD could be significantly decreased. This study indicated that a possible way to reduce the Internet gaming impulsivity of gamers with IGD is to increase the negativity of their implicit OEs with respect to Internet gaming.

## Data Availability Statement

The datasets generated for this study are available on request to the corresponding author.

## Ethics Statement

The studies involving human participants were reviewed and approved by the Beijing Normal University, China. The participants provided their written informed consent to participate in this study.

## Author Contributions

SH conceived of the presented idea, planned the experiments, and took the lead in writing the manuscript. XF provided funding acquisition, project administration, and resources. NZ performed the analytic calculations and contributed to the interpretation of the results. PC contributed to sample preparation and data collection.

## Funding

This study was supported by The Chinese Natural Science Foundation (Project No. 31170990) and Yangzi-River Professor Supportive Program of the corresponding author XF, and the Key Project of Shenzhen Education Science Plan in 2019 (zdzz19002) of the first author SH.

## Conflict of Interest

The authors declare that the research was conducted in the absence of any commercial or financial relationships that could be construed as a potential conflict of interest.
