# Supplementary material for: Effects of Increasing the Negativity of Implicit Outcome Expectancies on Internet Gaming Impulsivity
Source: Front Psychiatry. 2020 Apr 29;11:336. doi: 10.3389/fpsyt.2020.00336 (PMC7200976; doi:10.3389/fpsyt.2020.00336)
Supplement: Supplementary file 1 [file Table_1.docx]

Appendix 1. The stimulating words (internet gaming words and outcoming words) in the Go/No-go Association Task (GNAT)

| Internet gaming words | Direct positive (OE) | Direct negative (OE) | Indirect positive (OE) | Indirect negative (OE) |
| --- | --- | --- | --- | --- |
| 对战 (dui4 zhan4)  点卡 (dian3 ka3)  游戏币(you2 xi4 bi4)  Double Kill  打怪(da3 guai4)  等级(deng3 ji2)  战队(zhan4 dui4)  装备(zhuang1 bei4)  练级(lian4 ji2)  道具(dao4 ju4) | 升级了(sheng1 ji2 le)  爆出装备(bao4 chu1 Zhuang1 bei4)  满级(man3 ji2)  极品装备(ji2 pin3 zhuang1 bei4)  赢装备(ying2 zhuang1 bei4)  给经验(gei3 jing1 yan4)  PK赢(PK ying2)  给积分(gei3 ji1 fen1)  神器 (shen2 qi4)  打赢了(da3 ying2 le) | 自信感(zi4 xin4 gan3)  光荣感(guang1 rong2 gan3)  成就感(cheng2 jiu4 gan3)  快乐感(kuai4 le4 gan3)  刺激(ci4 ji)  娱乐的(yu2 le4 de)  被认可(bei4 ren4 ke3)  愉悦感(yu2 yue4 gan3)  满足感(man3 zu2 gan3)  高兴的(gao1 xing4 de) | 打输了(da4 shu1 le)  被虐了(bei4 nue4 le)  PK输(PK shu1)  输比赛(ying2 bi3 sai4)  减经验(jian3 jing1 yan4)  丢装备(diu1 zhuang1 bei4)  减积分(jian3 ji1 fen1)  被盗号(bei4 dao4 hao4)  交易被骗(jiao1 yi4 bei4 pian3)  职业削弱(zhi1 ye4 xue1 ruo4) | 情感淡漠(qing2 gan3 dan4 mo4)  学业差(xue2 ye4 cha4)  没前途(mei2 qian2 tu2)  健康差(jian4 kang1 cha4)  视力差(shi4 li4 cha4)  饮食差(yin2 shi2 cha4)  低落(di1 luo4)  身体差(shen1 ti1 cha4)  耽误正事(dan4 wu4 zheng4 shi4)  腰酸疼(yao1 suan1 teng2) |
